# Age-specific serum thyrotropin reference range for the diagnosis of subclinical hypothyroidism and its association with lipid profiles in the elderly population

**DOI:** 10.1038/s41598-022-24182-w

**Published:** 2022-12-03

**Authors:** Wenjing Ni, Mengjie Zhang, Xiaowei Wang, Xingjia Li, Qifeng Wang, Yan Wang, Guofang Chen, Tonggao Shen, Kuanlu Fan, Xiaoming Yao, Yu Sun, Chao Liu, Shuhang Xu

**Affiliations:** 1 Endocrine and Diabetes Center, The Affiliated Hospital of Integrated Traditional Chinese and Western Medicine, Nanjing University of Chinese Medicine, Jiangsu Province Academy of Traditional Chinese Medicine, Nanjing, 210028 China; 2 Key Laboratory of TCM Syndrome and Treatment of Yingbing (Thyroid Disease) of State Administration of Traditional Chinese Medicine, Jiangsu Province Academy of Traditional Chinese Medicine, Nanjing, China; 3 Department of Geriatrics, The Affiliated Suqian Hospital of Xuzhou Medical University, Suqian, China; 4 Suining Diabetes and Endocrine Hospital, Xuzhou, China; 5 Department of Endocrinology, The Second Affiliated Hospital of Xuzhou Medical University, Xuzhou, China; 6 Clinical Laboratory, Affiliated Hospital of Integrated Traditional Chinese and Western Medicine, Nanjing University of Chinese Medicine, Jiangsu Province Academy of Traditional Chinese Medicine, Nanjing, China; 7 Department of Endocrinology and Metabolism, The Affiliated Suqian Hospital of Xuzhou Medical University, Suqian, China

**Keywords:** Dyslipidaemias, Thyroid diseases

## Abstract

The overdiagnosis of subclinical hypothyroidism (SCH) in the elderly has driven researchers to establish age-specific thyroid stimulating hormone (TSH) intervals to precisely evaluate the prevalence of SCH. Moreover, abnormal lipid profiles, an insidious manifestation of SCH, show various impacts on different age groups. This study aimed to establish an age-specific TSH reference range to clarify the spectrum of SCH in the elderly. The prevalence of dyslipidemia and the age-specific association between TSH and lipid profiles were analyzed to elucidate the relationship between SCH and dyslipidemia. This cross-sectional study enrolled 2460 participants aged ≥ 65 years via cluster sampling. All participants received physical, laboratory tests and thyroid ultrasound examination and completed the questionnaire. The chi-square test was used to analyze variations of dyslipidemia prevalence among different groups. The Cochran-Armitage trend test was applied for testing the linear trends of age-specific prevalence of dyslipidemia among different TSH intervals in each age group. After adjusting for confounding factors, the age-specific association between TSH and lipid profiles was identified using multi-variate linear regression analysis. The TSH reference ranges in the 65–70 age group, 71–80 age group and > 80 age group were 0.65–5.51 mIU/L, 0.85–5.89 mIU/L and 0.78–6.70 mIU/L, respectively. Using these age-specific reference ranges, the prevalence of SCH in the whole population was 3.74%, which was significantly lower than the prevalence based on the laboratory reference range (10.28%). In the 65–70 age group, only the prevalence of high total cholesterol (TC) increased significantly with the age-specific TSH intervals, and TSH was positively associated with TC and low-density lipoprotein cholesterol (LDL-C). In the 71–80 and > 80 age groups, the prevalence of high TC, high triglycerides (TGs), and high LDL-C increased significantly with elevated TSH reference ranges. The levels of TC, TGs, and LDL-C were also positively associated with TSH level in 71–80 age group. However, such an association disappeared in > 80 age group. An age-specific reference range for TSH can effectively prevent the overdiagnosis of SCH in the elderly. Aging could somewhat attenuate the impact of TSH on lipid profiles.

## Introduction

Subclinical hypothyroidism (SCH), a highly prevalent thyroid disease, may be over-diagnosed among the elderly. Previous studies have showed that the prevalence of SCH was 1%-15% in people aged > 65 years worldwide^1^ and 35.22% in people aged ≥ 60 years in China^2^. Notably, thyroid stimulating hormone (TSH), the key indicator of SCH, is physiologically elevated with advanced age. Older people with mild increasing TSH levels above the upper limit of reference range may be misclassified as hypothyroid. Instead of applying TSH intervals provided by kit, age-specific TSH reference range grounded by United States National Academy of Clinical Biochemistry (NACB) guideline^3^ could evaluate the prevalence of SCH more precisely. Studies in Italy^4^, France^5^ and China^6–11^ have established regional TSH reference ranges for populations of different ages. However, some studies did not provide accurate urine iodine concentration (UIC) of the reference population, which had potential effect on TSH intervals establishment; others did not further stratify the elderly population, lacking specific reference range in different age groups. Therefore, it is necessary to establish a region- and age-specific TSH reference range for the elderly to accurately delineate the regional SCH spectrum.

It is well-known that elevated TSH can exacerbate lipid metabolism. A meta-analysis of 4 cohort studies in Europe has demonstrated a significant positive correlation between serum TSH within normal range (0.3–3.0 mIU/L) and triglycerides (TGs)^12^. The total cholesterol (TC) and TGs levels were found to be positively correlated with TSH level in healthy adults, while for SCH patients, their TGs level was significantly higher than the healthy population^13^. Interestingly, the association between TSH and serum lipid parameters varies among different age groups. Zhao et al.^14^ have reported that increased TSH levels were associated with elevated TC levels in people aged < 70 years, whereas such an association did not exist in the ≥ 70 age group, indicating the relationship among aging, TSH, and lipid profiles needs further investigation.

The present study aimed to establish age-specific TSH reference ranges among people aged ≥ 65 years to evaluate the prevalence of SCH. And the prevalence of dyslipidemia as well as the age-specific association between TSH and lipid profiles were also analyzed. Our findings may present the dynamic relationship between SCH and dyslipidemia in the elderly, hoping to provide some evidence for clinicians to guide surveillance and intervention in older patients with SCH, especially those with lipid abnormalities in the future.

## Results

### Population characteristics

A total of 2460 subjects were enrolled, with the average age 72.28 ± 5.85 years old, and average TSH 2.75 ± 4.49 mIU/L. The mean UIC of participants was 233.54 ± 66.64 μg/L. There were significant differences in age, height, weight, body mass index (BMI), TSH and TGs between three age groups (all *P* < 0.05) (Table 1).

**Table 1:** Population characteristics of 2460 elderly people.

	65–70 years (n = 1187)	71–80 years (n = 1012)	> 80 years (n = 261)	*P *value
Age (years)	67.45 ± 1.65	74.96 ± 2.91	83.87 ± 2.90	< 0.001
Height (m)	1.58 ± 0.08	1.57 ± 0.09	1.53 ± 0.10	< 0.001
Weight (kg)	63.01 ± 9.98	60.27 ± 10.77	56.15 ± 11.14	< 0.001
BMI (kg/m^2^)	25.17 ± 3.57	24.57 ± 3.77	24.10 ± 4.09	< 0.001
TSH (mIU/L)	2.61 ± 4.26	2.86 ± 5.05	2.95 ± 3.00	< 0.001
TPOAb (IU/mL)	26.29 ± 71.14	22.00 ± 56.89	26.52 ± 69.72	0.735
TgAb (IU/mL)	61.83 ± 245.93	61.44 ± 293.44	100.02 ± 453.43	0.765
UIC (µg/L)	230.21 ± 65.24	236.51 ± 67.79	237.20 ± 67.98	0.075
TC (mmol/L)	5.01 ± 1.03	4.94 ± 1.10	4.91 ± 0.97	0.121
TGs (mmol/L)	1.49 ± 1.15	1.37 ± 0.86	1.30 ± 0.69	0.006
LDL-C (mmol/L)	2.94 ± 0.83	2.91 ± 0.87	2.86 ± 0.78	0.212
HDL-C (mmol/L)	1.35 ± 0.38	1.37 ± 0.40	1.42 ± 0.39	0.076
HbA1c (%)	5.96 ± 1.11	6.01 ± 1.04	5.91 ± 0.98	0.229

BMI, body mass index; TSH, thyroid stimulating hormone; TPOAb, thyroid peroxidase; TgAb, thyroglobulin antibody; UIC, urine iodine concentration; TC, total cholesterol; TGs, triglycerides; LDL-C, low density lipoprotein cholesterol; HDL-C, high density lipoprotein cholesterol; HbA1c, glycosylated hemoglobin.

### Age-specific reference range of TSH

According to NACB, a total of 1074 elderly people were selected to determine the age-specific reference ranges of TSH (Supplementary Table 1). The 2.5th–97.5th percentile ranges for TSH were 0.65–5.51 mIU/L, 0.85–5.89 mIU/L and 0.78–6.70 mIU/L in 65–70 age group, 71–80 age group and > 80 age group, respectively (Table 2). The median level and upper limit of TSH increased with age.

**Table 2:** Age-specific reference range of TSH in three age groups (n = 1074).

Age (years)	n	TSH (mIU/L)		
		2.5th	50th	97.5th
65–70	532	0.65	2.06	5.51
71–80	439	0.85	2.27	5.89
> 80	103	0.78	2.66	6.70

TSH, thyroid stimulating hormone.

### Prevalence of SCH under 2 different reference ranges

As diagnosed by age-specific reference ranges, the prevalence of SCH in the whole population was 3.74%, which was significantly lower than the prevalence based on the laboratory reference range (3.74% vs. 10.28%, χ^2^ = 76.95, *P* < 0.001). Compared to the use of the common threshold of 4.20 mIU/L to diagnosis SCH, the prevalence of SCH based on the age-specific TSH reference ranges in three age groups were significantly decreased (65–70 age group: 3.62% vs. 8.76%, χ^2^ = 25.86; 71–80 age group: 3.85% vs. 11.17%, χ^2^ = 36.72; > 80 age group: 3.83% vs. 13.79%, χ^2^ = 15.49, all *P* < 0.001).

### Prevalence of dyslipidemia in different age groups

In the entire study population, the prevalence of dyslipidemia was 47.93% (1179/2460) and showed a significant downward trend with age (χ^2^ = 7.21, *P* = 0.027). The prevalence of high TGs significantly decreased with age (χ^2^ = 6.31, *P* = 0.043) while the prevalence of low HDL-C demonstrated a slightly downward tendency with age without statistical significance (χ^2^ = 2.31, *P* = 0.316, Table 3).

**Table 3:** Prevalence of dyslipidemia in three age groups (n = 2460).

Groups	Dyslipidemia	High TC	High TGs	High LDL-C	Low HDL-C
65–70 years	50.29% (597/1187)	11.29% (134/1187)	26.28% (312/1187)	7.08% (84/1187)	32.60% (387/1187)
71–80 years	46.74% (473/1012)	11.66% (118/1012)	22.92% (232/1012)	7.31% (74/1012)	29.84% (302/1012)
> 80 years	41.76% (109/261)*	8.81% (23/261)	19.92% (52/261)*	5.75% (15/261)	29.50% (77/261)

TC, total cholesterol; TGs, triglycerides; LDL-C, low density lipoprotein cholesterol; HDL-C, high density lipoprotein cholesterol.

*The linear trend of dyslipidemia prevalence was examined by the Cochran-Armitage trend test. Compared with 65–70 years group and 71–80 years group, *P* value < 0.05 was considered statistical significance.

Further, the prevalence of dyslipidemia stratified by different TSH intervals was analyzed in three age groups. The cutoffs of TSH were determined by the laboratory reference range and the upper limit of age-specific TSH reference range in each age group, respectively. The prevalence of high TC, high TGs and high LDL-C were all positively associated with TSH intervals in 71–80 age group and > 80 age group. Besides, the prevalence of dyslipidemia also rose with the increase of TSH in people aged 71–80 years, while a similar linear trend was observed in > 80 age group, but failed to reach statistical significance. In people aged 65–70 years, only the prevalence of high TC showed a significant upward linear trend with the increasing TSH (Table 4).

**Table 4:** Prevalence of dyslipidemia in elderly people stratified by age (n = 2460).

Groups	0.27 ≤ TSH ≤ 4.20	4.20 < TSH < ULN	≥ ULN	Linear trend (*P *value)
**65–70 years**^**a**^				
High TC	10.87% (117/1076)	15.00% (9/60)	18.18% (8/44)	0.038
High TGs	26.21% (282/1076)	28.33% (17/60)	27.27% (12/44)	0.758
High LDL-C	6.88% (74/1076)	10.00% (6/60)	9.09% (4/44)	0.143
Low HDL-C	33.55% (361/1076)	25.00% (15/60)	22.73% (10/44)	0.065
Dyslipidemia	39.13% (421/1076)	50.00% (30/60)	45.45% (20/44)	0.068
**71–80 years**^**b**^				
High TC	10.75% (96/893)	16.22% (12/74)	25.64% (10/39)	0.001
High TGs	22.17% (198/893)	22.97% (17/74)	41.03% (16/39)	0.021
High LDL-C	6.61% (59/893)	10.81% (8/74)	15.38% (6/39)	0.015
Low HDL-C	30.12% (269/893)	21.62% (16/74)	38.46% (15/39)	0.645
Dyslipidemia	45.58% (407/893)	48.65% (36/74)	61.54% (24/39)	0.031
**> 80 years**^**c**^				
High TC	7.66% (17/222)	11.54% (3/26)	20.00% (2/10)	0.032
High TGs	17.12% (38/222)	26.92% (7/26)	60.00% (6/10)	< 0.001
High LDL-C	4.50% (10/222)	7.69% (2/26)	20.00% (2/10)	0.018
Low HDL-C	29.28% (65/222)	34.62% (9/26)	30.00% (3/10)	0.655
Dyslipidemia	39.64% (88/222)	53.85% (14/26)	60.00% (6/10)	0.050

ULN of TSH for each group was 5.51mIU/L^a^, 5.89 mIU/L^b^, 6.70 mIU/L^c^, respectively.

ULN, upper limit of normal; TSH, thyroid stimulating hormone; TC, total cholesterol; TGs, triglycerides; LDL-C, low density lipoprotein cholesterol; HDL-C, high density lipoprotein cholesterol.

### The age-specific associations between TSH and lipid profiles

Multi-variate linear regressions were applied to investigate the association between TSH and lipid profiles. In the whole population, significant relationships were found between TSH and TC (*P* < 0.001), TGs (*P* = 0.001), and LDL-C (*P* < 0.001), but such relationships were not observed between TSH and HDL-C (*P* = 0.060) after adjusting for age, gender, height, weight, BMI and glycosylated hemoglobin (HbA1c). Specifically, each 1 mIU/L increase in TSH, the TC, TGs and LDL-C level tended to increase 0.0274 mmol/L, 0.0123 mmol/L and 0.0202 mmol/L, respectively. In addition, the association between TSH and lipid profiles were most prominent in 71–80 age group (all *P* < 0.001). In people aged 65–70 years, TSH only had significant positive relationships with TC (*P* = 0.003) and LDL-C (*P* = 0.001). However, the relationship between TSH and lipid profiles was weak in people aged more than 80 years (Table 5). The sex- and age-specific associations between TSH and lipid profiles exhibited no distinct differences compared with the results based on whole population stratified by age (Supplementary Table 2).

**Table 5:** Age-specific associations between TSH and lipid profiles in elderly people (n = 2460).

Age group (years)	Coefficient estimates^a^ (*P *value)^b^	Association of TSH with lipid profiles			
		TC (mmol/L)	TGs (mmol/L)	LDL-C (mmol/L)	HDL-C (mmol/L)
65–70	^β (*P* value)	0.021 0.003	0.005 0.525	0.019 0.001	0.004 0.127
71–80	^β (*P* value)	0.033 < 0.001	0.018 < 0.001	0.021 < 0.001	0.003 0.182
> 80	^β (*P* value)	0.024 0.203	0.016 0.234	0.028 0.066	− 0.004 0.625
Total	^β (*P* value)	0.027 < 0.001	0.007 0.001	0.020 < 0.001	0.003 0.060

TSH, thyroid stimulating hormone; TC, total cholesterol; TGs, triglycerides; LDL-C, low density lipoprotein cholesterol; HDL-C, high density lipoprotein cholesterol.

^a^Multiple linear regression analysis on the association between TSH and lipid profiles after adjusting for age, gender, height, weight, BMI and HbA1c.

^b^*P *value < 0.05 is considered statistically significant.

## Discussion

In this study, the age-specific TSH reference ranges for 65–70, 71–80 and > 80 age groups based on the NACB guideline were formulated. We found that the 50.0th and 97.5th percentiles of TSH increased with age. Studies from US^15^ and China^8^ showed that per 10-years increase in age was associated an elevation of 0.3 mIU/L and 0.534 mIU/L in the 97.5th percentile of TSH, respectively. Given the physiological adaptation of TSH with aging, modification of TSH reference range based on different age group is required. According to a nationwide study in China, the mean 2.5th and 97.5th percentiles of TSH were 2.28 mIU/L and 2.34 mIU/L among the whole population and the upper limit of TSH in the group aged ≥ 70 years was 10.50 mIU/L^8^ while in another Chinese study covering 10 cities showed that the upper limit of TSH in people aged ≥ 65 years was 8.86 mIU/L^7^, which were both higher than the thresholds established in this study. The diverse results in various studies may owe to the differences in sample size, regions, and iodine nutritional status. Because of the implementation of mandatory universal salt iodization, increased median UIC was positively associated with the 97.5th percentile of TSH but was negatively associated with the 2.5th percentile of TSH^8^. In a previous research, the TSH upper limit for the elderly with iodine excess was 6.423mIU/L in 60–69 years group and 11.520 mIU/L in ≥ 70 years group^11^, which were higher than the cut-off points for those with more than adequate iodine nutritional status in this study.

Regional- and age-specific TSH reference ranges may reduce the overdiagnosis of SCH in the elderly. In our present study, using the newly established TSH intervals, the prevalence of SCH was only 3.74%, which was significantly lower than that based on the laboratory criteria (10.28%). Zhai et al.^7^ reported that the prevalence of SCH remarkably declined (19.87% vs. 3.3%) when adopting the age-specific TSH intervals. In 2020, the French Society of Endocrinology recommended that the upper limit of TSH can be formulated as people’s age (decade) divided by 10 for the elderly over 60 years old^16^. Nevertheless, whether this recommendation is suitable in the older population in other countries or regions and the diagnostic efficiency of SCH based on this formulation needs further verification. The age-specific TSH reference ranges in our study may be more suitable for diagnosing older population who live in iodine-replete regions in China.

Based on the age-specific TSH reference ranges, we evaluated the prevalence of dyslipidemia and the association between TSH and lipid profiles among people of different TSH intervals in the three age groups. The results demonstrated significantly increased prevalence of high TC in all three age groups as the TSH intervals rose. In 71–80 and > 80 age groups, the rising TSH intervals were positive correlated with the prevalence of high TGs, high LDL-C, and dyslipidemia. These results indicated that lipid metabolism worsen with the elevated TSH level, which was parallel with some genome-wide association studies. They explored the genetic association between TSH and serum lipid profiles via Mendelian randomization (MR). According to Wang et al.^17^, per 1 standard deviation (SD) increase in normal TSH was significantly associated with a 0.048 SD increase in TC and a 0.032 SD increase in LDL-C, whereas TSH did not show a causal association with HDL-C and TGs. The study by van Vliet et al.^18^ unveiled a positive association between high TSH within normal range with the levels of very low-density lipoprotein subclasses and components, TGs, and the TG contents in lipoproteins.

This study also found the significant positive association between TSH and TC in 65–70 age group, and similarly, in 71–80 age group, TSH was also significantly positively associated with TC, TGs, and LDL-C. However, in people aged > 80 years, though there still existed such a positive association, it was not statistically significant. Moreover, the prevalence of dyslipidemia and high TGs exhibited a downward trend with age, which means aging may attenuate the impact of TSH on dyslipidemia in the elderly. Another Chinese study reported that in people aged 70 years and older, no association was observed between TSH and the serum lipid indicators^14^. According to the Sixth Korean National Health and Nutrition Examination Survey, though TSH was an independent risk factor for those with TC ≥ 200 mg/dL and LDL-C ≥ 130 mg/dL in the general female population, thyroid dysfunction was not correlated with lipid profiles in women older than 55 years^19^. It seems that the association between SCH and dyslipidemia changes by aging. In the older population, slight augment in the TSH level may not generate deleterious impact on lipid metabolism, compared with that in relatively young adults.

The advantage of this cross-sectional study is that instead of retrospectively collecting data in hospital, relatively healthy older population were recruited in this study, who can present a true spectrum of TSH values and prevalence of SCH in the elderly. We also further established age-specific TSH reference range for very old population. We admitted that this study has some limitations. Thyroid hormone levels were not measured, and therefore the effect of free thyroxine and triiodothyronine on peripheral lipid metabolism could not be adjusted. Also, other cofounding factors such as diet and lipid-lowering agents are not adjusted. Besides, the sample size of participants > 80 years was relatively small and the low statistical power potentially might weaken the age-specific relationship between TSH level and dyslipidemia to a certain degree. In the future, case–control and prospective cohort studies with larger sample size are required to further confirm the impact of aging on TSH, as well as the long-term effects of SCH on dyslipidemia. The indication of intervention for SCH-related diseases should also be explored to improve the management of thyroid and metabolic diseases in the elderly.

## Conclusions

This study shows that as the 97.5th percentile of TSH increases with age and age-specific TSH reference ranges can effectively avoid overdiagnosis of SCH in the elderly. A positive association between TSH and lipid profiles was observed. As aging, the lipid metabolism of the elderly deteriorates with the elevated age-specific TSH reference ranges, whereas the very old age may attenuate the effect of TSH on lipid profiles. The role of aging in the association between TSH and lipid metabolism needs more explorations.

## Materials and methods

### Study design and population

Thyroid diseases in Older Population: Screening, Surveillance and Intervention (TOPS) study was a cross-sectional, population-based study, which was carried out from May to October 2021. It was approved by the Ethics Committee of Affiliated Hospital of Integrated Traditional Chinese and Western Medicine, Nanjing University of Chinese Medicine. All methods were performed in accordance with the relevant guidelines and regulations. Participants who lived in two rural areas in Jiangsu Province of China were selected via cluster sampling. People were eligible if they aged 65 years or older, lived in local residence for at least five years. People who had cognitive dysfunction or other mental disorders were excluded. All subjects signed the informed consent.

**Figure 1:**
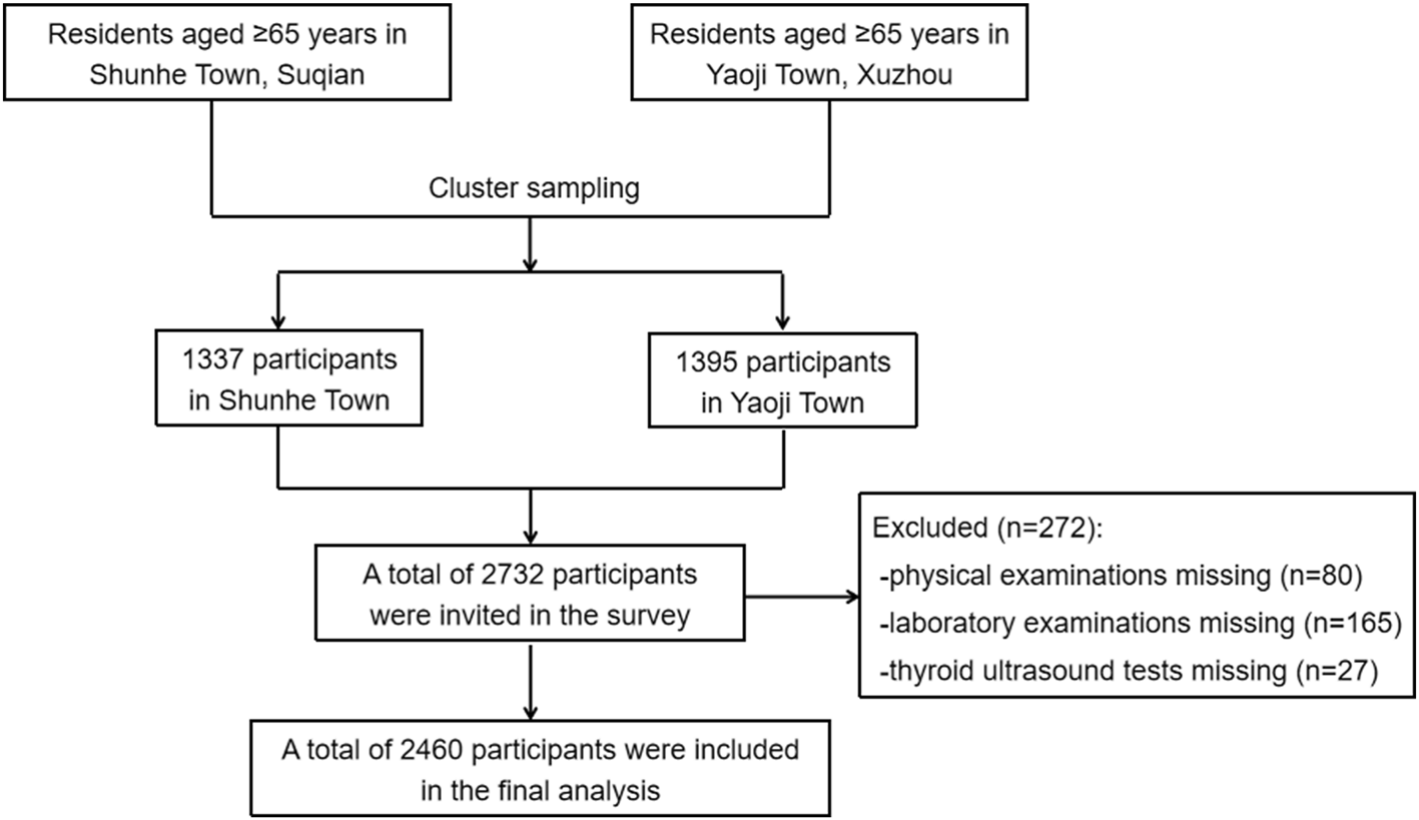
The flow chart.

Participants received physical and thyroid ultrasound examination, and completed the questionnaire including demographic characteristics, personal and family history of thyroid and metabolic diseases, medication and previous medical history. A total of 2460 people with complete information were finally enrolled in this study (Fig. 1). The whole population was stratified by three groups according to their chronological age: 65–70 years (1187 people), 71–80 years (1012 people), > 80 years (261 people).

The NACB guideline^3^ was applied to define age-specific reference ranges of TSH. In this cohort, 1074 people were selected to calculate reference ranges, and they all met the following criteria: 1) with no personal or family history of thyroid dysfunction; 2) with negative thyroid autoantibodies (thyroid peroxidase antibody [TPOAb] and thyroglobulin antibody [TgAb]); 3) with no goiter and thyroid nodules detected by ultrasound; 4) not on any medications (except estrogen) that influence thyroid function. The comparisons between the NACB cohort and the whole population and the unselected population were performed and presented in the Supplementary Table 3 and Supplementary Table 4, respectively.

### Measurements and examinations

Fasting blood and urine samples were collected between 8:00 a.m. and 10:00 a.m. All samples were transported under -20℃ to the Laboratory Department for measurement. In this study, free thyroxine (FT4) was measured only if the TSH level was outside the normal laboratory reference range.

TSH, FT4, TPOAb and TgAb were measured by electrochemiluminescence method. TC, TGs, LDL-C, HDL-C and UIC were measured by colorimetry. The above indexes were all tested by Swiss Roche Cobas 702 biochemical analyzer. HbA1c was determined by high pressure liquid chromatography via Bio-Rad Bole D-10 glycated hemoglobin instrument. In our center, the laboratory reference ranges of TSH, FT4, TPOAb and TgAb were 0.27–4.20 mIU/mL, 12.0–22.0 pmol/L, < 115.00 IU/mL and < 34.00 IU/mL, respectively.

All participants were maintained in a supine extension to fully expose the neck skin and keep smooth breathing. Two experienced radiologists performed thyroid ultrasound examination. SIUI Shantou Apogee 1000 color Doppler ultrasound diagnostic instrument was used with a probe frequency of 7.5–13.0 MHz.

### Diagnostic criteria

SCH was defined as TSH higher than the upper limit of the normal reference range and FT4 within the normal range^20^. According to the National Cholesterol Education Program Adult Treatment Panel III criteria (NCEP/ATPIII)^21^, dyslipidemia was defined by meeting any of the following criteria: (a) high TC: TC ≥ 6.22 mmol/L; (b) high TGs: TGs ≥ 1.70 mmol/L; (c) high LDL-C: LDL-C ≥ 4.14 mmol/L; (d) low HDL-C: HDL-C < 1.04 mmol/L for men and < 1.30 mmol/L for women.

The iodine nutritional status of elderly people was assessed using the standards of WHO/UNICEF/ICCIDD: < 99 μg/L for iodine deficiency, 100–199 μg/L for adequate iodine nutrition, 200–299 μg/L for more than adequate iodine nutrition, and > 300 μg/L for excessive iodine nutrition^22^.

### Statistical analysis

The R programming language was used for statistical analyses. All descriptive data were presented as the mean ± standard deviation. Parametric or nonparametric tests were applied to examine the normality of data distribution. Continuous variables in normal distribution were tested by Student’s t-test and non-normally distributed data including age, TSH, TPOAb, TgAb, HbA1c and TGs was analyzed by the Mann–Whitney U test. The chi-square test was used to analyze variations of dyslipidemia prevalence among different groups. The Cochran-Armitage trend test was applied for analyzing the linear trends for age-specific prevalence of dyslipidemia among different TSH intervals in each age group. The age-specific associations between TSH and lipid profiles were tested by multi-variate linear regression analysis. *P* < 0.05 was considered as statistically significant.

### Ethics

This study was approved by the Ethics Committee of Affiliated Hospital of Integrated Traditional Chinese and Western Medicine, Nanjing University of Chinese Medicine.

### Consent to participate

All subjects signed the written informed consent.

### Consent for publication

All of the authors approved the publication of the article.

## Supplementary Information

Supplementary Tables.

## Data Availability

The datasets generated and analyzed in the present study are available from the corresponding author upon reasonable request.
