# Supplementary material for: Age-specific serum thyrotropin reference range for the diagnosis of subclinical hypothyroidism and its association with lipid profiles in the elderly population
Source: Sci Rep. 2022 Dec 3;12:20872. doi: 10.1038/s41598-022-24182-w (PMC9719481; doi:10.1038/s41598-022-24182-w)
Supplement: Supplementary file 1 — Supplementary Tables. [file 41598_2022_24182_MOESM1_ESM.docx]

**Supplementary Table 1**. Population characteristics of elderly people meeting NACB criteria (n=1074)

|  | Total  (n=1074) | Male  (n=668) | Female  (n=406) | *P-*value |
| --- | --- | --- | --- | --- |
| Age (years) | 72.03±5.68 | 71.90±5.63 | 72.24±5.77 | 0.404 |
| Height (m) | 1.58±0.08 | 1.62±0.07 | 1.52±0.06 | <0.001 |
| Weight (kg) | 61.29±10.90 | 64.05±10.91 | 56.75±9.24 | <0.001 |
| BMI (kg/m^2^) | 24.52±3.76 | 24.46±3.83 | 24.62±3.63 | 0.419 |
| TSH (mIU/L) | 2.49±1.37 | 2.44±2.32 | 2.69±1.47 | <0.001 |
| TPOAb (IU/mL) | 10.12±5.38 | 9.84±5.29 | 10.63±5.56 | 0.008 |
| TgAb (IU/mL) | 15.12±11.00 | 14.79±10.97 | 15.65±11.02 | 0.005 |
| UIC (µg/L) | 234.06±69.43 | 234.98±64.47 | 232.67±76.94 | 0.469 |
| TC (mmol/L) | 4.90±1.06 | 4.74±0.97 | 5.18±1.14 | <0.001 |
| TGs (mmol/L) | 1.37±0.93 | 1.26±0.89 | 1.57±0.96 | <0.001 |
| LDL-C (mmol/L) | 2.90±0.85 | 2.81±0.80 | 3.07±0.90 | <0.001 |
| HDL-C (mmol/L) | 1.38±0.37 | 1.37±0.37 | 1.40±0.36 | 0.193 |
| HbA1c (%) | 5.93±1.05 | 5.88±1.03 | 6.00±1.07 | 0.010 |

NACB, National Academy of Clinical Biochemistry; BMI, body mass index; TSH, thyroid stimulating hormone; TPOAb, thyroid peroxidase; TgAb, thyroglobulin antibody; UIC, urine iodine concentration; TC, total cholesterol; TGs, triglycerides; LDL-C, low density lipoprotein cholesterol; HDL-C, high density lipoprotein cholesterol; HbA1c, glycosylated hemoglobin.

**Supplementary Table 2**. Age-specific associations between TSH and lipid profiles in elderly men and women (n=2460)

| Age group  (years) | Coefficient estimates ^a^  (*P-*value) ^b^ | the association of TSH with lipid profiles | | | |
| --- | --- | --- | --- | --- | --- |
|  |  | TC  (mmol/L) | TGs  (mmol/L) | LDL-C  (mmol/L) | HDL-C  (mmol/L) |
| Men (n=1251) |  |  |  |  |  |
| 65-70 years | ^β  (*P*-value) | 0.016  0.013 | 0.004  0.657 | 0.014  0.012 | 0.003  0.257 |
| 71-80 years | ^β  (*P*-value) | 0.041  <0.001 | 0.001  0.854 | 0.029  <0.001 | 0.006  0.063 |
| >80 years | ^β  (*P*-value) | -0.005  0.920 | 0.004  0.924 | 0.048  0.251 | -0.014  0.449 |
| Total | ^β  (*P*-value) | 0.025  <0.001 | 0.003  0.551 | 0.020  <0.001 | 0.004  0.057 |
| Women (n=1209) |  |  |  |  |  |
| 65-70 years | ^β  (*P*-value) | 0.054  0.020 | 0.019  0.421 | 0.06  0.001 | 0.009  0.233 |
| 71-80 years | ^β  (*P*-value) | 0.028  0.002 | 0.029  <0.001 | 0.015  0.043 | 0.001  0.748 |
| >80 years | ^β  (*P*-value) | 0.030  0.155 | 0.173  0.221 | 0.026  0.121 | -0.002  0.841 |
| Total | ^β  (*P*-value) | 0.031  <0.001 | 0.027  <0.001 | 0.021  <0.001 | 0.001  0.584 |

^a^ Multiple linear regression analysis on the association between TSH and lipid profiles after adjusting for age, gender, height, weight, BMI and HbA1c.

^b^ *P-*value <0.05 was considered statistically significant.

TSH, thyroid stimulating hormone; TC, total cholesterol; TGs, triglycerides; LDL-C, low density lipoprotein cholesterol; HDL-C, high density lipoprotein cholesterol.

**Supplementary Table 3.** The comparison between the NACB cohort and the whole population

|  | the NACB cohort  (n=1074) | The whole population  (n=2460) | P-value |
| --- | --- | --- | --- |
| Age (years) | 72.03±5.68 | 72.28±5.85 | 0.352 |
| Height (m) | 1.58±0.08 | 1.57±0.09 | <0.001 |
| Weight (kg) | 61.29±10.90 | 61.16±10.65 | 0.854 |
| BMI (kg/m2) | 24.52±3.76 | 24.81±3.73 | 0.023 |
| TSH (mIU/L) | 2.49±1.37 | 2.75±4.49 | 0.468 |
| TPOAb (IU/mL) | 10.12±5.38 | 24.55±65.50 | <0.001 |
| TgAb (IU/mL) | 15.12±11.00 | 65.72±294.03 | <0.001 |
| UIC (µg/L) | 234.06±69.43 | 233.54±66.64 | 0.993 |
| TC (mmol/L) | 4.90±1.06 | 4.97±1.06 | 0.041 |
| TGs (mmol/L) | 1.37±0.93 | 1.42±1.00 | 0.090 |
| LDL-C (mmol/L) | 2.90±0.85 | 2.92±0.84 | 0.574 |
| HDL-C (mmol/L) | 1.38±0.37 | 1.37±0.39 | 0.275 |
| HbA1c (%) | 5.93±1.05 | 5.98±1.07 | 0.811 |

NACB, National Academy of Clinical Biochemistry; BMI, body mass index; TSH, thyroid stimulating hormone; TPOAb, thyroid peroxidase; TgAb, thyroglobulin antibody; UIC, urine iodine concentration; TC, total cholesterol; TGs, triglycerides; LDL-C, low density lipoprotein cholesterol; HDL-C, high density lipoprotein cholesterol; HbA1c, glycosylated hemoglobin.

**Supplementary Table 4.** The comparison between the NACB cohort and the unselected cohort

|  | the NACB cohort  (n=1074) | the unselected cohort  (n=1386) | P-value |
| --- | --- | --- | --- |
| Age (years) | 72.03±5.68 | 72.48±5.96 | 0.107 |
| Height (m) | 1.58±0.08 | 1.56±0.09 | <0.001 |
| Weight (kg) | 61.29±10.90 | 61.12±10.79 | 0.793 |
| BMI (kg/m2) | 24.52±3.76 | 25.06±3.77 | <0.001 |
| TSH (mIU/L) | 2.49±1.37 | 2.95±5.86 | 0.246 |
| TPOAb (IU/mL) | 10.12±5.38 | 35.75±85.51 | <0.001 |
| TgAb (IU/mL) | 15.12±11.00 | 104.93±387.13 | <0.001 |
| UIC (µg/L) | 234.06±69.43 | 233.14±64.42 | 0.987 |
| TC (mmol/L) | 4.90±1.06 | 5.02±1.05 | 0.001 |
| TGs (mmol/L) | 1.37±0.93 | 1.45±1.05 | 0.007 |
| LDL-C (mmol/L) | 2.90±0.85 | 2.93±0.84 | 0.369 |
| HDL-C (mmol/L) | 1.38±0.37 | 1.35±0.40 | 0.081 |
| HbA1c (%) | 5.93±1.05 | 6.01±1.09 | 0.384 |

NACB, National Academy of Clinical Biochemistry; BMI, body mass index; TSH, thyroid stimulating hormone; TPOAb, thyroid peroxidase; TgAb, thyroglobulin antibody; UIC, urine iodine concentration; TC, total cholesterol; TGs, triglycerides; LDL-C, low density lipoprotein cholesterol; HDL-C, high density lipoprotein cholesterol; HbA1c, glycosylated hemoglobin.
